# Determination and pharmacokinetics study of UK-5099 in mouse plasma by LC–MS/MS

**DOI:** 10.1186/s12917-022-03245-0

**Published:** 2022-04-20

**Authors:** Qingyuan Zeng, Hongfei Si, Kun Lv, Jiao Mo, Xinnian Wang, Biqing Yan, Jili Zhang

**Affiliations:** 1 Intensive Care Unit, The Affiliated Hospital of Medical School, Ningbo University, Ningbo, China; 2 Ningbo University School of Medicine, Ningbo University, Ningbo, China; 3 College of Pharmacy, Jinan University, Guangzhou, China; 4 Ningbo University School of Business, Ningbo University, Ningbo, China; 5 Lanzhou Institute of Husbandry and Pharmaceutical Sciences, Chinese Academy of Agricultural Sciences, Lanzhou, 315211 China

**Keywords:** UK-5099, HPLC–MS/MS, Mouse plasma, Pharmacokinetics, Glibenclamide

## Abstract

**Background:**

UK-5099 is a potent mitochondrial acetone carrier inhibitor, that exhibits anticancer activity. Recently, the anti-*Toxoplasma gondii* activity of UK-5099 was proposed, and in vivo studies of its pharmacokinetics in BALB/c mice are necessary to further evaluate the clinical effect of UK-5099.

**Methods and results:**

A simple and fast high-performance liquid chromatography-tandem mass spectrometry (HPLC–MS/MS) analysis method was established and verified in terms of its linearity, matrix effect, accuracy, precision, recovery and stability. The analytes were separated by an Agilent ZORBAX XDB-C18 column (2.1 × 50 mm, 3.5 μm) at 30 °C. A gradient mobile phase consisting of water with 0.1% formic acid (FA) (phase A) and acetonitrile (ACN) (phase B) was delivered at a flow rate of 0.40 mL·min^−1^ with an injection volume of 5 μL. A good linear response was obtained in a concentration range of 5–5000 ng·mL^−1^ (*r*^2^ = 0.9947). The lower limit of quantification (LLOQ) was 5 ng·mL^−1^. The extraction recovery of UK-5099 was greater than 95%. The inter- and intra-day accuracy and precision of the method showed relative standard deviations (RSDs) of less than 15%. This method has been successfully applied to the pharmacokinetic evaluation of UK-5099 in mouse plasma. In health mice, the main pharmacokinetic parameters of UK-5099 after intraperitoneal administration were measured using a noncompartmental model, in which the AUC_0-t_ was 42,103 ± 12,072 ng·h·mL^−1^ and the MRT_0-t_ was 0.857 ± 0.143 h. The peak concentration (C_max_) was 82,500 ± 20,745 ng·h·mL^−1^, which occurred at a peak time (T_max_) = 0.250 ± 0.000 h.

**Conclusions:**

A fast and sensitive HPLC–MS/MS method was developed, validated and successfully used for the determination of UK-5099 levels in mice after intraperitoneal administration. This study was the first report of the pharmacokinetic parameters of UK-5099 in mice, which will help to further study the administration of UK-5099 in animals and humans.

## Introduction

UK-5099 (alpha-cyano-beta-(1-phenylindol-3-yl) acrylate), an alpha-cyanocinnamate derivative, which is a potent mitochondrial acetone carrier inhibitor [1, 2]. It could inhibit the mitochondrial acetone carrier (MPC) by binding to C54 of MPC2 in a covalent reversible manner with a low dissociation rate of the inhibitor-carrier complex, which assists the transport of pyruvate across the inner mitochondrial membrane, plays a key role in the metabolism of carbohydrates, lipids and amino acids, and then blocks the entry of pyruvate into mitochondria, weakening mitochondrial oxidative phosphorylation (OXPHOS) and triggering aerobic glycolysis [1, 3–5]. Compared with parental cells, UK-5099-treated tumor cells also showed stronger invasive ability [6], and a significant decrease in tumor pHe of 0.22 units in UK-5099 treated mice [7]. Moreover, UK-5099 inhibited the oxidation of pyruvate in rat heart mitochondria in a nonlinear inhibitory kinetic manner [2]. In glucagon-treated rats, UK-5099 inhibited pyruvate carboxylation and total pyruvate metabolism in a linear relationship [8]. In rat liver and heart mitochondria, UK-5099 inhibited the consumption of pyruvate-dependent 0_2_ with an IC_50_ value of 50 nM [4]. UK-5099 could be used to treat hair loss, showing good efficacy and promoting obvious hair growth in mice [9, 10]. Furthermore, UK-5099 (Ki = 49 mM) completely blocked the trypanosomal pyruvate carrier and killed trypanosomes [11]. In addition, treatment with the MPC inhibitor, UK5099 increased the levels of the glycolytic enzymes HK2, PFKFB3, and LDHA, promoting glycolysis and lactate secretion in human umbilical vein endothelial cells [12]. A previous study also observed that human colon organoid colony formation and growth were promoted by UK-5099 treatment [13].

Recently, we also found that UK-5099 has anti-*Toxoplasma gondii* activity. Thus, in vivo studies of its pharmacokinetics are necessary to evaluate the clinical effects of UK-5099. Therefore, it is necessary to evaluate the pharmacokinetics of UK-5099 in mice.To date, no specific pharmacokinetic research on UK-5099 has been reported. The purpose of this study was to establish a sensitive and reliable LC–MS/MS method to analyze the pharmacokinetics of UK-5099 in mouse plasma, and to study its application by examining the pharmacokinetics of UK-5099 in mice.

## Results

### Mass spectrometry and liquid chromatography

**Fig. 1:**
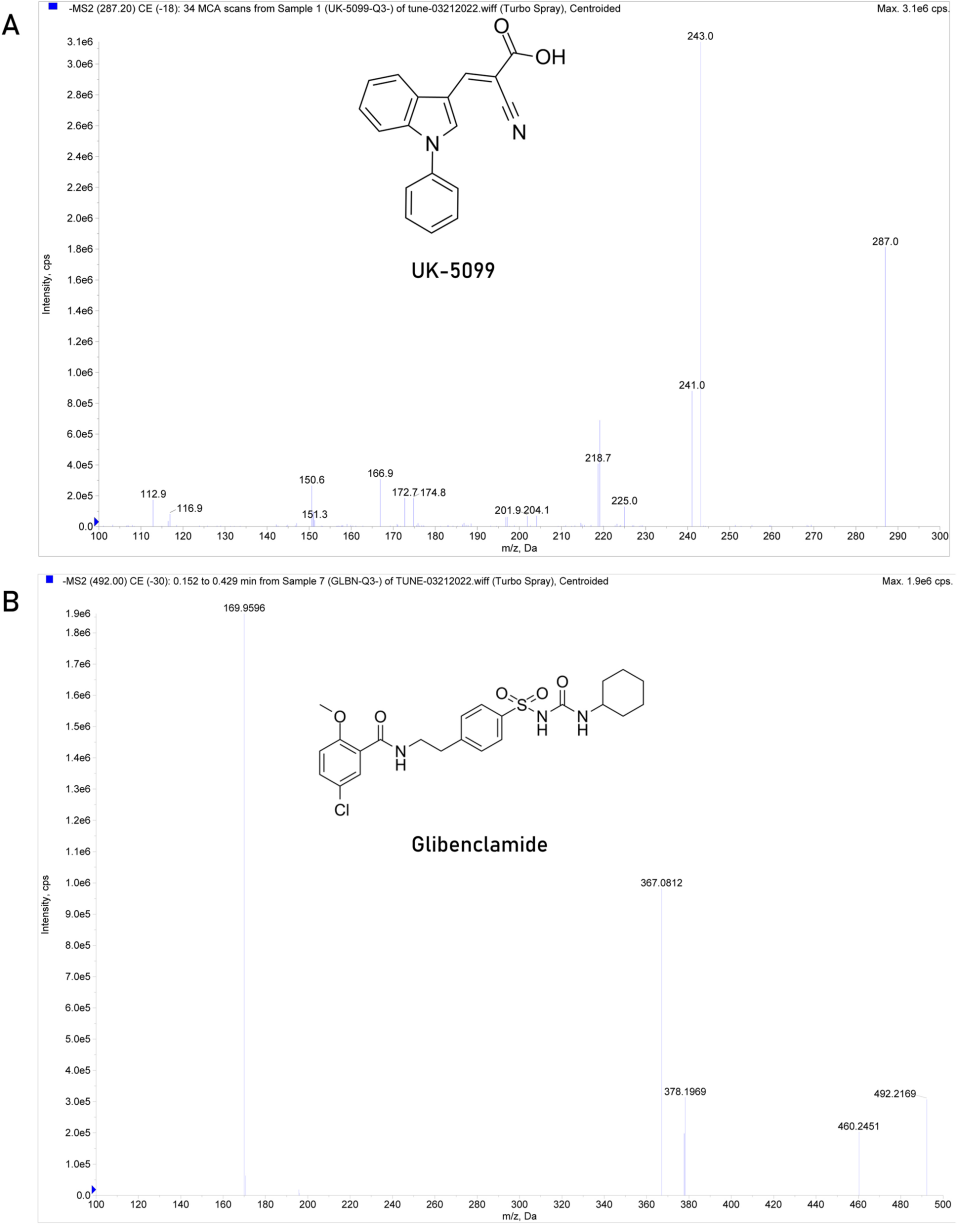
Full-scan product ion spectra of [M-H]^−^ ions for UK-5099 (**A**) and IS (**B**)

In the negative electrospray ionization (ESI) mode, UK-5099 showed a good response. The full-scan ion spectrum indicated that the most abundant ions observed in UK-5099 were m/z 287.2 and m/z 492.0 of the internal standard (IS). The quantification of UK-5099 was carried out in multiple reaction monitoring (MRM) mode to obtain highly selective and sensitive data. The mass transitions chosen for quantitation were m/z 287.2 to 243.0 for UK-5099 and m/z 492.0 to 169.9 for IS (Fig. 1).

Mobile phase A (water with 0.1% FA), mobile phase B (ACN with 0.1% FA) and a 50 mm chromatographic column were used for flow elution at a flow rate of 0.4 mL·mL^−1^ for 3 min, and chromatographic separation was carried out. Under optimized LC and MS conditions, the separation retention times of UK-5099 and IS were 1.39 min and 1.40 min, respectively. Endogenous substances in plasma did not interfere with the detection of analytes.

### Method validation

#### Selectivity and matrix effects

**Fig. 2:**
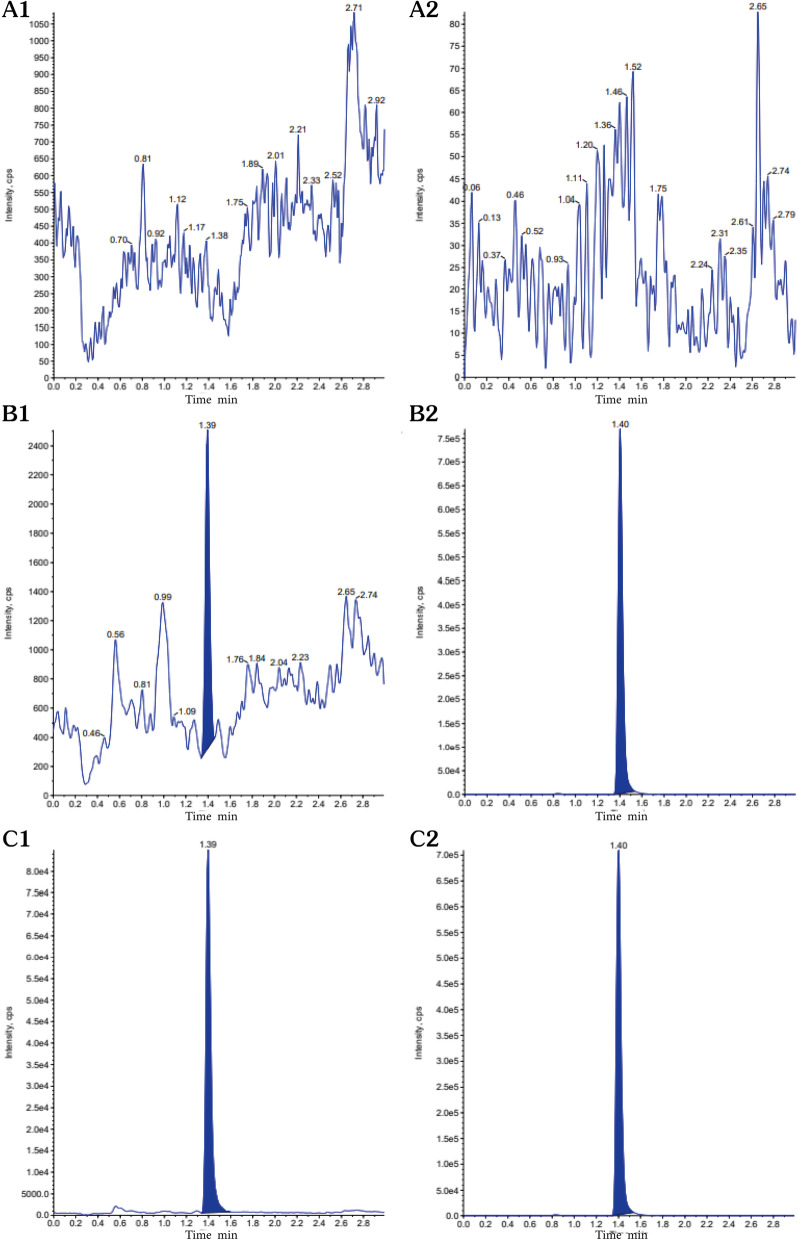
Representative chromatograms of UK-5099 and the IS in plasma. **A** Blank plasma; (**B**) a blank plasma sample spiked with the LLOQ; (**C**) a plasma sample obtained after oral administration of 20 mg.kg^−1^ UK-5099

The selectivity of the method was evaluated by analyzing blank plasma samples from nine different mice under the chromatographic conditions described above. Figure 2 shows representative chromatograms of blank plasma, LLOQ (5 ng·mL^−1^) plasma samples and test samples collected after intraperitoneal administration. The results showed that the determination was not subject to interference mediated by endogenous substances and was close to the retention time of UK-5099 or IS.

By evaluating the effect of the plasma matrix on the levels of UK-5099 and IS in 9 different blank mouse plasma samples, the average matrix effect on UK-5099 was 98.81% ± 1.44%, and the matrix effect on IS was 99.53 ± 2.97%. The results showed that the plasma matrix and the method did not significantly interfere with the mobile relative measurement.

#### LLOQ and linearity

**Fig. 3:**
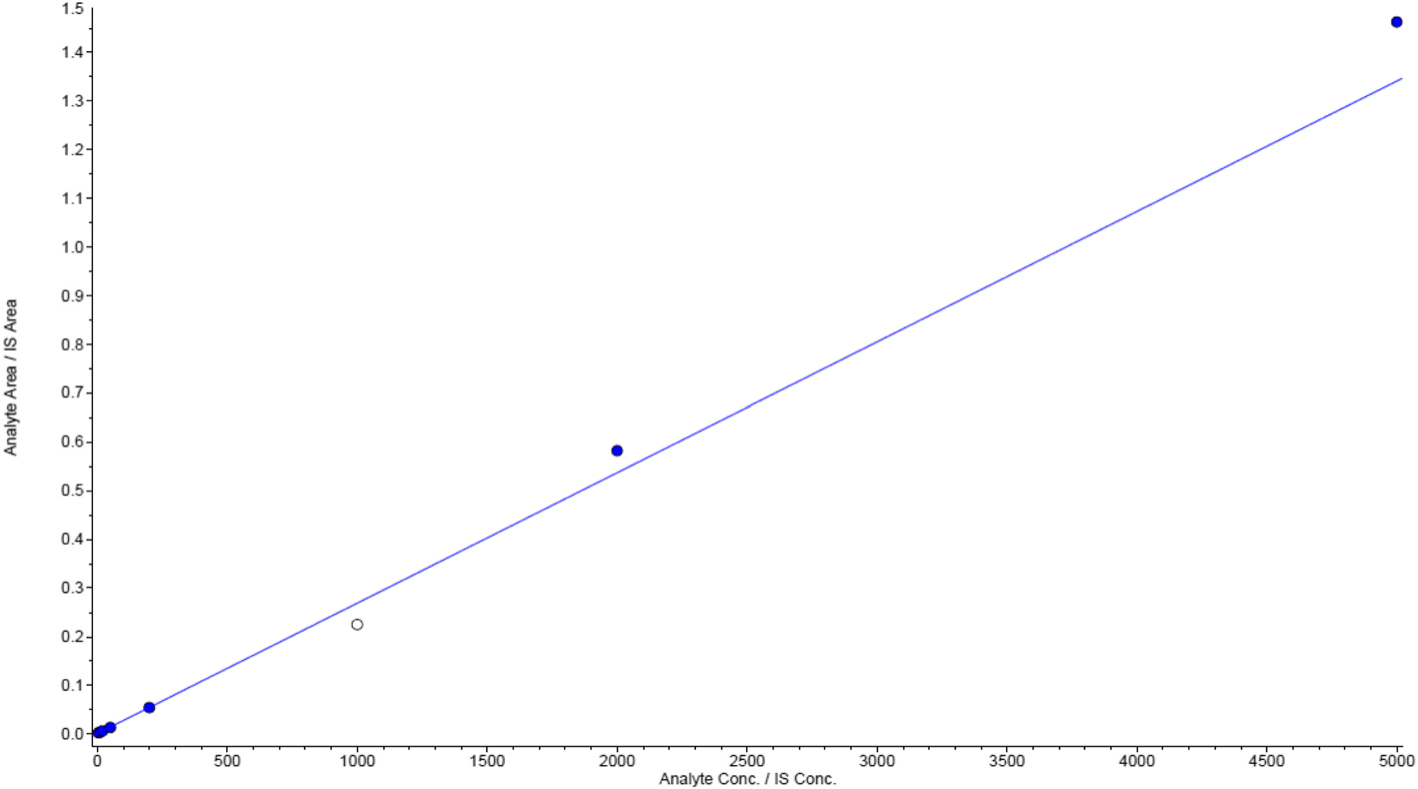
Calibration curves

The LLOQ and lower limit of detection (LLOD) of UK-5099 were 5 ng·mL^−1^ and 3 ng·mL^−1^, respectively. According to the results of the weighted (1/x^2^) least squares linear regression analysis, the calibration curve of UK-5099 was linear in the concentration range of 5–5000 ng·mL^−1^. The extrapolation equation of the calibration curve of UK-5099 was y = 0.000293x + 0.00112 (*r*^2^ = 0.9947) (Fig. 3).

#### Accuracy and precision

Table 1 summarized the intra- and inter-day precision and accuracy based on QC samples (15 ng·mL^−1^、400 ng·mL^−1^、4000 ng·mL^−1^) and LLOQ samples (5 ng·mL^−1^). The intra- and inter-day accuracy were 96.67–110.25% and 95.33–111.75%, respectively. The intra-day and inter-day precisions were 0.50–12.81% and 4.31–10.47%, respectively. The results showed that the LC–MS/MS method used in this study had good precision and accuracy.

**Table 1:** Intra- and inter-day precision and accuracy of the determination of UK-5099 in mouse plasma

Concentration (ng/mL)	Intra-day precision and accuracy (*n* = 6)		Inter-day precision and accuracy (*n* = 18)	
	Accuracy (%) ± SD	RSD (%)	Accuracy (%) ± SD	RSD (%)
5	98.48 ± 4.90	4.98	103.85 ± 5.83	5.61
15	96.67 ± 0.50	0.50	95.33 ± 4.80	5.04
400	110.25 ± 14.70	12.81	111.75 ± 11.70	10.47
4000	109.25 ± 1.70	1.67	104.50 ± 4.50	4.31

#### Recovery

Table 2 showed the recovery rate of UK-5099 extracted from the plasma matrix. The average extraction recovery rates of UK-5099 at QC concentration (15 ng·mL^−1^、400 ng·mL^−1^、4000 ng·mL^−1^) were 91.11 ± 5.18%, 111.75 ± 4.75% and 111.79 ± 8.16%, respectively. The average recovery rate of IS was 109.37 ± 4.91%.

**Table 2:** Recovery of UK-5099 (*n* = 6) from mouse plasma

Concentration (ng/mL)		Recovery (%, *n* = 6)	
		Mean (%) ± SD	RSD (%)
UK-5099	15	91.11 ± 5.18	5.68
	400	111.75 ± 4.75	4.25
	4000	111.79 ± 8.16	7.30
IS		109.37 ± 4.91	4.49

#### Stability

Table 3 showed the stability results of UK-5099 under different storage conditions. The accuracy was 95.11–115.47%, and the precision (RSD%) was 2.26–10.96%, indicating that UK-5099 was quite stable under all of the experimental conditions.

**Table 3:** Stability of UK-5099 in mouse plasma samples under various conditions (*n* = 6)

Storage conditions	Concentration (ng/mL)	Accuracy ± SD (%)	RSD (%)
Ambient temperature for 24 h	15	115.47 ± 7.11	6.16
	400	107.42 ± 4.65	4.65
	4000	106.67 ± 3.53	3.31
At -20 °C for 60 days	15	107.73 ± 9.12	8.47
	400	108.75 ± 5.00	4.60
	4000	106.22 ± 6.01	5.66
At 4 °C in the autosampler for 24 h	15	95.11 ± 10.42	10.96
	400	107.42 ± 4.65	4.33
	4000	104.08 ± 5.30	5.09
Three Freeze–thaw cycles	15	99.11 ± 6.71	6.77
	400	108.75 ± 5.00	4.60
	4000	100.33 ± 2.27	2.26

#### Dilution integrity

The plasma concentration of UK-5099 after intraperitoneal administration exceeded the linear range. Therefore, the dilution effect on UK-5099 was studied. The accuracy and precision of ten-fold diluted plasma samples (*n* = 6) were 98.81% and 6.73, respectively.

### Pharmacokinetic studies

**Fig. 4:**
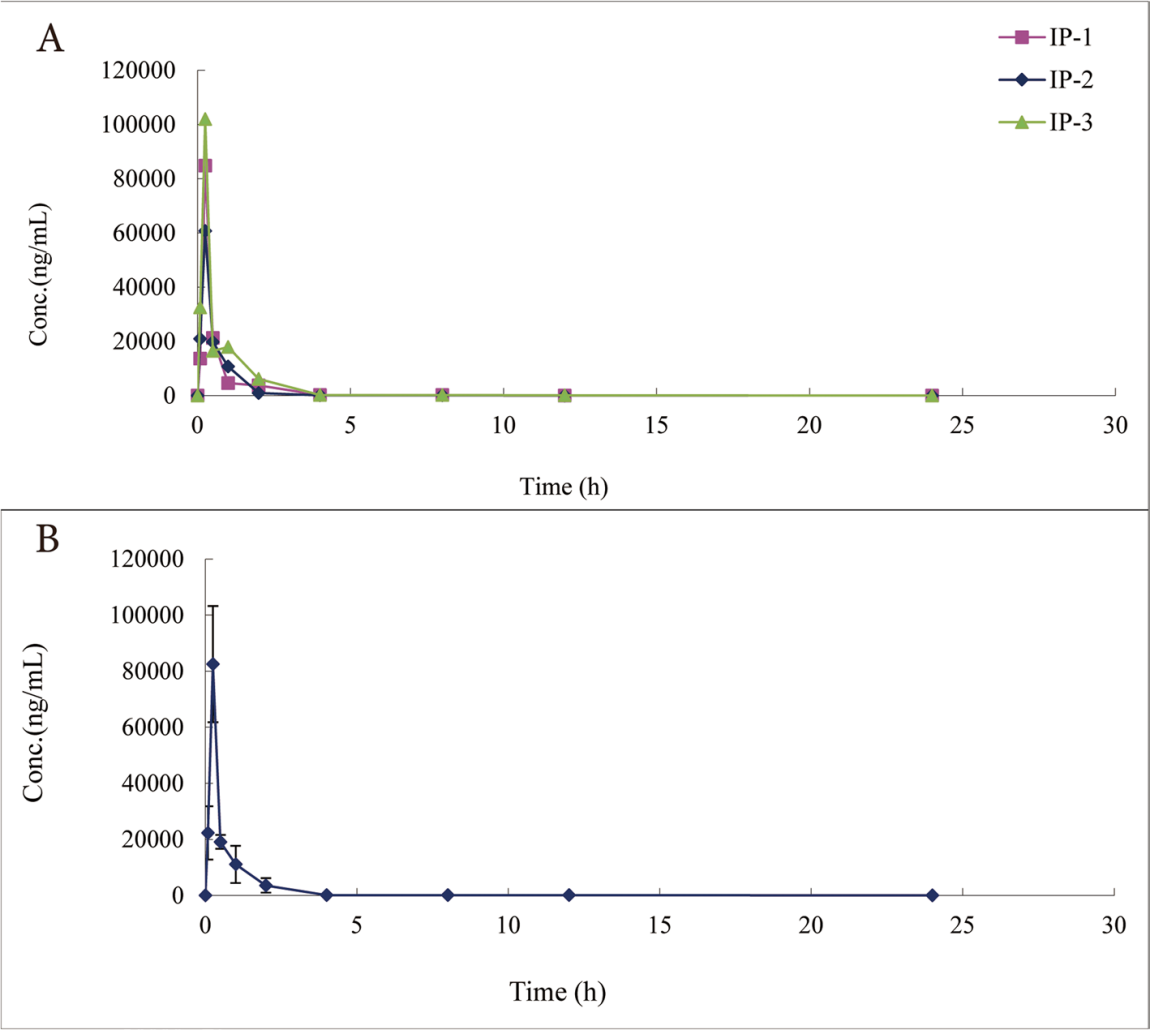
Mean plasma concentration–time profile after the administration of UK-5099 to mice (*n* = 3)

After intraperitoneal injection at a dose of 20 mg/kg, the plasma concentration of UK-5099 was determined. The average plasma concentration–time curve of UK-5099 was shown in Fig. 4. In addition, the main pharmacokinetic parameters of UK-5099 after intraperitoneal administration were determined using a noncompartmental model and were listed in Table 4. The C_max_ of UK-5099 in mice was 82,500 ± 20,745 ng·mL^−1^, the T_max_ was 0.250 ± 0.000 h, the AUC_0-t_ was 42,103 ± 12,072 h·ng^−1^·mL^−1^, and the MRT_0-t_ was 0.857 ± 0.143 h.

**Table 4:** The pharmacokinetic parameters of UK-5099 in mice following oral or intraperitoneal administration (*n* = 3)

Parameters (Units)	Mean ± SD
K_el_ (h^−1^)	0.052 ± 0.160 13.358 ± 0.239
T_1/2_ (h)	
T_max_ (h)	0.250 ± 0.000
C_max_ (ng/mL)	82,500 ± 20,745
AUC_0-t_ (h/ng/mL)	42,103 ± 12,072
AUC_0-inf_ (h/ng/mL)	44,088 ± 12,098
MRT_0-t_ (h)	0.857 ± 0.143
MRT_0-inf_ (h)	2.227 ± 0.155

## Discussion

Considering the characteristics of UK-5099, to improve the ionization degree of the compound and the peak area in the method development stage, FA and ACN were employed in the mobile phase, and the composition of the mobile phase was adjusted to be suitable for the separation and ionization of UK-5099, with good peak shape and resolution. Furthermore, the LC–MS/MS method was validated in terms of its selectivity, linearity, matrix effect, LLOQ, recovery, accuracy and precision and stability according to guidelines established by the US Food and Drug Administration for bioanalytical method validation [14]. Subsequently, we established a good linear relationship between the UK-5099 concentration and the quantitative ion peak area ratio. In addition, the sample preparation process in the experiment was simple and rapid, there was no interference of endogenous substances within the retention time, and UK-5099 remained stable in mouse plasma under different storage conditions. Furthermore, the intra- and inter-day precision and accuracy of the method were within acceptable ranges, indicating that the LC–MS/MS method developed in this study was reliable and repeatable in the quantitative analysis of UK-5099 levels in mouse plasma. Therefore, the LC–MS/MS method developed and verified in our study was simple and sensitive, and could be used to determine the pharmacokinetics of UK-5099 in plasma.

In our previous study, UK-5099 exerted good anti-*Toxoplasma gondii* activity, but the pharmacokinetics in vivo have not been reported (Data not yet published). In this study, after intraperitoneal administration 20 mg·kg^−1^ UK-5099, the maximum plasma concentration reached 82,500 ± 20,745 ng·mL^−1^, which reached the effective concentration for the treatment of *Toxoplasma gondii*. Furthermore, it could quickly reach the maximum blood concentration in the mouse, while the T_max_ was 0.250 h. What's more exciting was that the half-life (T_1/2_) was very long, up to 13.358 ± 0.239 h. Moreover, the AUC_0-t_ (h·ng^−1^·mL^−1^) was 42,103 ± 12,072 h·ng^−1^·mL^−1^. Accordingly, UK-5099 showed good pharmacokinetic characteristics for the development of anti-*Toxoplasma gondii* drugs. However, the influence of other modes of administration on its pharmacokinetics needs to be further explored.

## Conclusions

A simple and rapid LC–MS / MS method was developed in this study. In this method, the sample preparation method was simple, and the analytical method had good linearity, sensitivity, precision and accuracy. This method was successfully used to determine the pharmacokinetics of UK-5099 in plasma after intraperitoneal administration in mice. This study reported the pharmacokinetic parameters of UK-5099 in mice after intraperitoneal injection for the first time, which may contribute to the pharmacodynamic study of UK-5099 in animals and humans.

## Materials and methods

### Reagents

Standard UK-5099 and Glibenclamide (internal standard, IS) were provided by Selleck. Co., Ltd. (USA) (batch numbers S894301 and S171603, respectively, purities > 99.8%). Analytical grade formic acid (FA) and acetonitrile (ACN) were purchased from Fisher Chemical Company (Waltham, MA, USA). Water was purified through a Milli-Q Plus water system (Millikonre, Bedford, Massachusetts, USA) before use.

### LC/MS/MS analysis

The UPLC–MS/MS instrument (AB SCIEX Triple QuadTM 5500) consisted of an LC system with a binary pump (Model SL) and a triple-quadrupole mass spectrometer with an ESI interface. The analytes were separated on an Agilent ZORBAX XDB-C18 column (3.5 μm, scale 2.1 × 50 mm). The mobile phase was consisted of water with 0.1% formic acid (FA) (phase A) and acetonitrile (ACN) with 0.1% FA (phase B), which was delivered at a flow rate of 0.4 mL·min^-1^, and the following gradient profile was used: (1) 0–0.50 min, 50% B; (2) 0.50–1.00 min, 50%-98% B; (3) 1.00–1.90 min, 98% B; (4) 1.91 min, 50% B; and (5) 3.00 min, stop. The sample was injected at a volume of 5 μL at 30 °C. Mass spectrometry was performed by a triple-quadrupole mass spectrometer and ESI under negative ion mode. MassHunter software was used for data acquisition (Analysist 1.6.3, AB Sciex).

### Preparation of standard solutions and working solutions

As the stock solution, UK-5099 was diluted in ACN/water (1:1, v: v) to 50 μg·mL^−1^. Calibration standards were prepared by diluting the corresponding standard working solutions with blank mouse plasma to achieve final concentrations of 5.00, 10.0, 20.0, 50.0, 200, 1000, 2000, 5000 ng·mL^−1^. Quality control (QC) samples with concentrations of 5 ng·mL^−1^ (LLOQ), 15 ng·mL^−1^ (LQC, low quality control), 400 ng·mL^−1^ (MQC, medium quality control) and 4000 ng·mL^−1^ (HQC, high quality control) were prepared with the stock solution by dilution. One hundred milligrams of IS was dissolved with methanol in a 100 mL brown volumetric flask to produce an IS stock solution with a concentration of 1000 μg·mL^−1^. The IS stock solution was further diluted with ACN to prepare the working solution (50 ng·mL^−1^). All of the solutions were stored at 4 °C and brought to room temperature before use [15].

### Sample preparation

An aliquot of 10 μL of sample was added to 200 μL of ACN containing IS (Glibenclamide, 50 ng·mL^−1^) vortexed for 10 min, and then centrifuged at 3000 rpm for 8 min. Then, 60 μL of supernatant was added to 120 μL of water, and vortexed for 10 min. An equal 5 μL volume of the mixture was injected into the LC–MS/MS system.

### Method validation

The LC–MS/MS method was used to verify the selectivity, linearity, matrix effect, LLOQ, recovery, accuracy, precision, and stability.

#### Selectivity and matrix effects

Blank plasma samples from 9 different mice were analyzed to confirm that there were no interference peaks and to assess the selectivity of interference.

The matrix effect was evaluated by comparing the area response of a blank plasma sample upon adding UK-5099 after extraction at three QC levels and an equivalent concentration standard solution adding the same mobile phase [16].

#### LLOQ and linearity

The LLOQ and LLOD were determined as the ratio of signal to baseline noise (S/N) and wereat least 10 and 3, respectively. The calibration curve of UK-5099 was linear in the concentration range of 5 to 5000 ng. mL^−1^. The calibration curve was constructed by plotting the relationship between the peak area ratio of UK-5099 vs. IS (y) and the nominal concentration of UK-5099 (x) (y = ax + b), and the least square method was used for linear regression analysis. A correlation coefficient (r^2^) of at least 0.99 was required to meet the standard.

#### Accuracy and precision

Precision was considered the relative standard deviation (RSD) of repeated measurements, and accuracy was determined by calculating the ratio of the concentration to the theoretical concentration. The intra-day accuracy and precision of the HPLC–MS/MS method were determined by analyzing the quality control concentration and the 6 repeated LLOD concentrations of each concentration on the same day. The inter-day accuracy and precision were evaluated by analyzing the QC and LLOD concentrations in 6 replicates of each concentration over 3 days [17]. According to ICH, the standard of precision and accuracy was RSD ≤ 15% for each concentration, except for LLOQ (≤ 20%) [18].

#### Recovery and stability

The recovery rate was determined by comparing the analysis results of the extracted QC samples with the unextracted pure standard samples. QC samples (15 ng·mL^−1^, 400 ng·mL^−1^ and 4000 ng·mL^−1^) were evaluated, and each concentration was repeated 6 times to evaluate the efficiency of extracting UK-5099 from the biological matrix.

The stability was evaluated by analyzing repeats (*n* = 6) of QC samples with concentrations of 15 ng·mL^−1^, 400 ng·mL^−1^ and 4000 ng·mL^−1^ under different sample storage and processing procedures: (1) plasma samples were kept at ambient temperature for 24 h (2) plasma samples were stored at -20 °C for 60 days (3) plasma samples were stored in an autosampler at 4 °C for 24 h (4) plasma samples were measured after three freeze–thaw cycles (25 °C to -20 °C) [19].

#### Dilution integrity

To study the dilution integrity of UK-5099 in plasma we accurately quantified UK-5099 was accurately quantified at concentrations exceeding the maximum value of the calibration curve. Plasma samples (*n* = 6) at concentrations ranging from 10,000 to 1000 ng/mL were diluted ten-fold with blank plasma. The diluted samples were further quantified according to the calibration curve. The accuracy and precision of the determination after dilution should be within the acceptable limit (RSD%, 15%) [20].

### Pharmacokinetic study in mice

Nine BALB/c female mice (Animal Quality Certificate No.: 20200916Abzz619000305) were obtained from Vitonlihua Experimental Animal Technology Ltd, with a body weight (BW) of 16.8–20.0 g, and were adapted to the standard environmentally controlled animal room (25 ± 2 °C, relative humidity 50%, 12:12 h light/dark cycle) for 1 week. All mice were raised under almost the same conditions and were provided sufficient water and food. The mice were fasted overnight before administration to eliminate the impact of food on drug absorption, and the mice resumed eating 4 h after administration [17]. During the experiment, the mice could freely drink water. After the experiment, the mice were moved into the carbon dioxide death room, anesthetized with sevoflurane and euthanized with carbon dioxide. As gently as possible to minimize the pain of the mice. After the mice were no longer moving breathing, they were confirmed dead after another two minutes of observation.

UK-5099 was dissolved in corn oil with 10% DMSO at a concentration of 2 mg·mL^−1^ for intraperitoneal injection. UK-5099 was injected intraperitoneally into mice at a dose of 20 mg·kg^−1^ based on live body weight. All mice were raised under almost the same conditions and provided sufficient water and food. Blood samples (100 μL) were collected at 0 h, 0.083 h, 0.25 h, 0.5 h, 1 h, 2 h, 4 h, 8 h, 12 h, and 24 h. After all blood samples were centrifuged at 3000 rpm for 10 min, plasma samples were collected and immediately stored at -20 °C until analysis. The experimental protocol was approved by the institutional ethics committee of the Lanzhou Institute of Husbandry and Pharmaceutical Sciences, China (No.: SCXK (GAN) 2020–0005). All of the experimental methods, the animal care and the barn environment of this study were approved and implemented in accordance with the guidelines for the care and use of experimental animals of the Lanzhou Institute of Animal Science and Veterinary Pharmacy. The study was carried out in compliance with the ARRIVE guidelines.

WinNonlin professional software version 8.0 (Pharsight, Mountain View, California, USA) was used to calculate the pharmacokinetic parameters. The optimal pharmacokinetic model was determined according to the minimum Akaike Information Criterion (AIC) value principle, and used for data fitting and parameter estimation. The area under the plasma curve (AUC), the peak plasma concentration (C_max_), the half-life (T_1/2_) and the peak time (T_max_) were expressed as the mean ± SD.

## Data Availability

The datasets generated during and analyzed during the current study are not publicly available due to the data as part of new drug application, but are available from the corresponding author on reasonable requests.

## Abbreviations

AUC_0-t_: Area under the concentration–time curve from time zero to the last time point
BW: Body weight
C_max_: Peak plasma concentration
ESI: Electrospray ionization
HPLC–MS/MS: High-performance liquid chromatography-tandem mass spectrometry
IS: Internal standard
LC–MS/MS: Liquid chromatography coupled with tandem mass spectrometry
LLOD: Lower limit of detection
LLOQ: Lower limit of quantification
MRM: Multiple reaction monitoring
QC: Quality control
RSD: Relative standard deviation
T_max_: Peak time
T_1/2_: Half-life
FA: Formic acid
ACN: Acetonitrile
LQC: Low quality control
MQC: Medium quality control
HQC: High quality control
r^2^: Coefficient of correlation
AIC: Akaike Information Criterion
AUC: Area under the plasma curve

## References

[1] Proudlove MO, Beechey RB, Moore AL (1987). Pyruvate transport by thermogenic-tissue mitochondria. Biochem J.

[2] Halestrap AP (1975). The mitochondrial pyruvate carrier Kinetics and specificity for substrates and inhibitors. Biochem J.

[3] Simchowitz L (1988). Properties of the principal anion-exchange mechanism in human neutrophils. Soc Gen Physiol Ser.

[4] Zhong Y, Li X, Yu D, Li X, Li Y, Long Y (2015). Application of mitochondrial pyruvate carrier blocker UK5099 creates metabolic reprogram and greater stem-like properties in LnCap prostate cancer cells *in vitro*. Oncotarget.

[5] Yamashita Y, Vinogradova EV, Zhang X, Suciu RM, Cravatt BF (2020). A chemical proteomic probe for the mitochondrial pyruvate carrier complex. Angew Chem Int Ed Engl.

[6] Li Y, Li X, Kan Q, Zhang M, Li X, Xu R (2017). Mitochondrial pyruvate carrier function is negatively linked to Warburg phenotype in vitro and malignant features in esophageal squamous cell carcinomas. Oncotarget.

[7] Buyse C, Joudiou N, Corbet C, Feron O, Mignion L, Flament J (2021). Impact of inhibition of the mitochondrial pyruvate carrier on the tumor extracellular pH as measured by CEST-MRI. Cancers..

[8] Halestrap AP, Armston AE (1984). A re-evaluation of the role of mitochondrial pyruvate transport in the hormonal control of rat liver mitochondrial pyruvate metabolism. Biochem J.

[9] Liu X, Flores AA, Situ L, Gu W, Ding H, Christofk HR (2021). Development of novel mitochondrial pyruvate carrier inhibitors to treat hair loss. J Med Chem.

[10] Flores A, Schell J, Krall AS, Jelinek D, Miranda M, Grigorian M (2017). Lactate dehydrogenase activity drives hair follicle stem cell activation. Nat Cell Biol.

[11] Wiemer EA, Michels PA, Opperdoes FR (1995). The inhibition of pyruvate transport across the plasma membrane of the bloodstream form of Trypanosoma brucei and its metabolic implications. Biochem J.

[12] Wang D, Wang Q, Yan G, Qiao Y, Zhu B, Liu B (2018). Hypoxia induces lactate secretion and glycolytic efflux by downregulating mitochondrial pyruvate carrier levels in human umbilical vein endothelial cells. Mol Med Rep.

[13] Schell JC, Wisidagama DR, Bensard C, Zhao H, Wei P, Tanner J (2017). Control of intestinal stem cell function and proliferation by mitochondrial pyruvate metabolism. Nat Cell Biol.

[14] Food and Drug Administration of the United States. Guidance for Industry—Bioanalytical Method Validation; US Department of Health and Human Services, Center for Drug Evaluation and Research (CDER), Center for Veterinary Medicine (CVM): Rockville, USA. 2001.

[15] Wang X, Chen M, Wen C, Zhang Q, Ma J (2013). Determination of chidamide in rat plasma by LC-MS and its application to pharmacokinetics study. Biomedical chromatography: BMC.

[16] Singh A, Thatikonda T, Kumar A, Wazir PVV, Nandi U, Singh PP (2018). Determination of ZSTK474, a novel Pan PI3K inhibitor in mouse plasma by LC-MS/MS and its application to Pharmacokinetics. J Pharm Biomed Anal.

[17] Li B, Gong SY, Zhou XZ, Yang YJ, Li JY, Wei XJ (2017). Determination of antibacterial agent tilmicosin in pig plasma by LC/MS/MS and its application to pharmacokinetics. Biomed Chromatogr..

[18] Baber N (2012). International conference on harmonisation of technical requirements for registration of pharmaceuticals for human use (ICH). British J Clin Pharmacol.

[19] Zhang JL, Si HF, Sun JC, Lv K, Yan BQ, Li B (2021). Determination of myrislignan levels in BALB/c mouse plasma by LC-MS/MS and a comparison of its pharmacokinetics after oral and intraperitoneal administration. BMC Vet Res.

[20] Zhang JL, Bai YB, Li B, Zhou XZ, Si HF, Zhang JY (2019). Determination and pharmacokinetics study of oxyclozanide suspension in cattle by LC-MS/MS. BMC Vet Res.

